# From One-Size-Fits-All to Data-Driven Recovery: A Narrative Review of Wearable Technologies Toward Personalized Rehabilitation After Total Hip and Knee Arthroplasty

**DOI:** 10.3390/jpm16070393

**Published:** 2026-07-22

**Authors:** Stefano Pagano, Sebastian Dendorfer, Tobias Renkawitz

**Affiliations:** 1 Department of Orthopaedics, University of Regensburg, Asklepios Klinikum Bad Abbach, Kaiser-Karl V.-Allee 3, 93077 Bad Abbach, Germany; 2 Laboratory of Biomechanics, Ostbayerische Technische Hochschule Regensburg, Seybothstraße 2, 93053 Regensburg, Germany

**Keywords:** total hip arthroplasty, total knee arthroplasty, wearable technology, digital phenotyping, personalized rehabilitation

## Abstract

**Background/Objectives**: Commercial wearables are increasingly used after total hip and knee arthroplasty (THA/TKA) to record activity and gait outside the clinic and may help tailor recovery pathways. We reviewed the evidence on commercial devices, digital phenotyping, and data-informed rehabilitation after arthroplasty. **Methods**: We performed a structured narrative search of PubMed and Google Scholar. Of 916 records, 63 duplicates were removed, 853 were screened, 245 full texts were assessed, and 76 publications were included. Findings were synthesized thematically; no formal risk-of-bias assessment or evidence grading was undertaken. **Results**: Recovery differed by outcome and procedure. PROMs commonly improved within 1–3 months, while gait-quality measures recovered over about 7–13 weeks after THA and 13–24 weeks after TKA. Frequency-domain gait features, sample entropy, and machine-learning models provided information beyond step counts. Tracker-based interventions most consistently increased postoperative steps when introduced early and combined with behavior-change support, while digital rehabilitation was generally non-inferior to conventional rehabilitation. Wrist-worn consumer devices remained inaccurate with gait aids, compliance definitions varied, and higher step counts did not consistently correspond to better PROMs. **Conclusions**: Commercial wearables already support phenotyping and remote follow-up of individual recovery trajectories, but evidence for fully adaptive rehabilitation remains limited.

## 1. Introduction

Rehabilitation after total hip and knee arthroplasty (THA/TKA) has traditionally been organized around standardized postoperative milestones, fixed exercise programs, and intermittent clinic-based assessments. Commercial wearable technologies challenge this model by providing continuous, real-world information on how patients recover at home, including walking volume, cadence, gait symmetry, speed, stair use, and joint motion [1,2,3,4,5,6,7].

Accordingly, this review deliberately focuses on commercially available consumer and clinical-grade systems rather than laboratory prototypes, because these technologies are closest to routine clinical implementation, patient self-monitoring, reimbursement discussions, and scalable postoperative care pathways.

This shift is not simply technological; it reflects a broader movement toward digital phenotyping, in which passively collected behavioral and movement data are used to characterize recovery at the level of the individual patient rather than the average cohort [8]. In arthroplasty, this distinction is important because subjective improvement often precedes objective functional normalization, and because THA and TKA follow different recovery trajectories that may require different monitoring strategies [1,9,10,11,12,13].

The aim of this review is to examine how commercial wearables are being used after THA/TKA, how far the field has progressed from simple activity tracking to digital phenotyping, whether wearable-supported rehabilitation improves outcomes, and which technical, methodological, and clinical barriers continue to limit implementation in personalized care [1,2,14,15,16,17].

## 2. Evidence Identification and Scope

A structured literature search was conducted in PubMed and Google Scholar to identify studies evaluating the use of commercially available wearable technologies and smart devices in postoperative rehabilitation following THA/TKA.

In PubMed, a comprehensive search strategy combined Medical Subject Headings (MeSH) and free-text terms related to arthroplasty, rehabilitation, and digital monitoring technologies. The full PubMed query was: ((“Arthroplasty, Replacement, Hip”[Mesh] OR “Arthroplasty, Replacement, Knee”[Mesh] OR “total hip arthroplasty”[tiab] OR THA[tiab] OR “total hip replacement”[tiab] OR “total knee arthroplasty”[tiab] OR TKA[tiab] OR “total knee replacement”[tiab]) AND (“Rehabilitation”[Mesh] OR rehabilitation[tiab] OR recovery[tiab] OR postoperative[tiab] OR post-operative[tiab] OR telerehabilitation[tiab] OR “remote monitoring”[tiab]) AND (“Wearable Electronic Devices”[Mesh] OR wearable*[tiab] OR smartwatch*[tiab] OR “smart watch”[tiab] OR Fitbit[tiab] OR “Apple Watch”[tiab] OR smartphone*[tiab] OR acceleromet*[tiab] OR gyroscop*[tiab] OR “activity tracker*”[tiab] OR mHealth[tiab] OR “mobile health”[tiab] OR sensor*[tiab])).

Google Scholar was used only to supplement PubMed with recent or cross-disciplinary studies that were not indexed there. Candidate records were assessed against the same eligibility criteria.

Records were eligible when they involved adult THA or TKA patients, focused on postoperative rehabilitation or recovery, used commercially available wearable or smart-device technologies, reported objective or quantifiable outcomes, and were available as full-text peer-reviewed publications. Studies were excluded if they addressed non-orthopaedic populations, non-postoperative settings, experimental or non-commercial sensor systems, or lacked objective outcome data. Conference abstracts, protocols, and editorials were not treated as primary source evidence.

**Figure 1:**
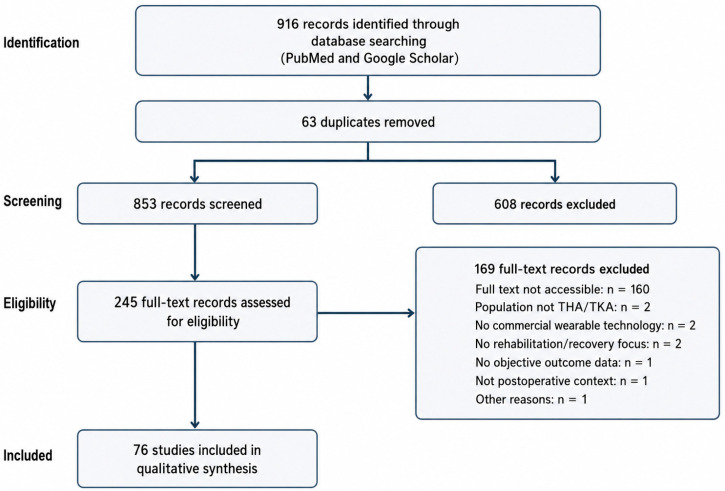
Study selection workflow of the source evidence base.

The combined search returned 916 records. After 63 duplicates were removed, 853 titles and abstracts were screened and 608 records were excluded. Of 245 full texts assessed, 169 were excluded; 160 of these could not be accessed. The final narrative synthesis included 76 publications (Figure 1). These comprised primary studies, evidence syntheses, validation studies, and selected implementation papers. Broader digital-health reports and protocols were used only for context.

We extracted device type, methods used for digital phenotyping, recovery trajectories, approaches to personalization, comparative effectiveness, and implementation issues. The thematic sections below constitute the narrative synthesis. No study was excluded on the basis of methodological quality, and the review did not include formal risk-of-bias assessment, quantitative pooling, or evidence grading.

## 3. Characteristics of the Included Literature

The 76 publications included reviews, meta-analyses, cohort and pilot studies, observational gait-sensor work, validation studies, and randomized or quasi-randomized trials. Classification by stated population or scope identified 43 knee-specific publications, 7 hip-specific publications, 19 that addressed both procedures or total joint arthroplasty, and 7 broader contextual sources. The predominance of TKA literature is therefore substantial. Study sizes ranged from fewer than 20 participants in mechanistic analyses to hundreds or thousands in multicenter and platform-based cohorts [9,10,11,12,13,18,19,20,21]. Follow-up ranged from postoperative day 1 [22] to 6–12 months [9,10,13,23].

Direct comparison remains difficult because device class, sensor placement, outcome definitions, wear-time thresholds, analytic methods, and rehabilitation settings differ across studies. The synthesis therefore separates hip and knee findings when procedure-specific data are available and uses mixed or contextual sources for cross-cutting themes (Table 1).

## 4. Commercial Wearable Technologies in Arthroplasty Rehabilitation

### 4.1. Consumer-Grade and Clinical-Grade Devices

The device landscape spans everyday consumer platforms and purpose-built clinical systems. Among consumer devices, Fitbit models, Garmin activity trackers, Apple Watch, iPhone-based sensing, Withings monitors, Nokia Go, and app-linked smartwatch ecosystems appear most frequently [3,9,10,11,12,14,20,26,29,32,33,34,35,36,37,62,63,65,72,73]. Their chief advantage is scalability: they are inexpensive relative to clinical platforms, familiar to patients, and well suited to continuous real-world monitoring of activity volume and selected gait metrics [1,33].

Clinical-grade and rehabilitation-specific systems, including TracPatch Duo, GaitSmart, APDM Opal, SENS Motion, Vicon Blue Trident, Xsens MTw Awinda, MotionSense, BoostFix, and eCEN Care, occupy a different role [1,39,40,41,42,44,45,46,47,50,51,52,55,56,70,74]. These systems usually integrate accelerometers and gyroscopes, and sometimes magnetometers, within dedicated inertial measurement unit (IMU) platforms. Compared with wrist-worn consumer devices, they offer richer biomechanical data, including symmetry, cadence structure, knee motion, and movement quality, but at higher cost and with greater setup demands [1,18,24,44,50,51].

Across both categories, accelerometry remains the core sensing modality. Smartphone-embedded sensors increasingly provide a lower-cost route to three-dimensional motion capture, while smart implants represent the most advanced end of the spectrum by generating passive step data from within the prosthesis environment [20,21]. The literature therefore reflects a hierarchy of tools with different trade-offs between convenience, signal fidelity, and clinical specificity [1,2,4,5]. These device-level differences are clinically relevant because they determine which recovery dimensions can be measured reliably and which signals can plausibly support individualized rehabilitation decisions.

### 4.2. Sensor Placement and Measurement Consequences

Sensor location matters because it determines which movement features can be measured accurately. Wrist-worn devices are convenient, but they perform poorly during the early postoperative period when patients use walkers or crutches. In the most direct validation study, Apple Watch Series 4 and Fitbit Charge showed acceptable agreement without gait aids but clinically unacceptable bias while patients walked with a walker [64]. This limitation is important because the period when complications and mobility setbacks are most clinically relevant overlaps with the period when consumer wrist devices are least reliable [64].

By contrast, thigh-, ankle-, tibial-, trunk-, and knee-mounted IMUs have been used to capture stride time, duty factor, asymmetry, impact loading, symmetry indices, knee flexion-extension, and other biomechanical features that are difficult to infer accurately from the wrist [39,42,43,44,47,48,50,53,56,57]. Smartphone placement in pockets or bags introduces additional noise, whereas embedded or surface-mounted systems reduce placement variability and provide more targeted signals [20,21]. These observations support a simple principle: device choice should be driven by the intended clinical question rather than by convenience alone [27].

## 5. Digital Phenotyping and Recovery Trajectories

### 5.1. From Step Counts to Multidimensional Gait Biomarkers

Early arthroplasty studies focused mainly on daily step count and global physical activity [33,75]. Those measures were useful because they offered objective, scalable alternatives to patient-reported outcomes, yet they captured only one dimension of recovery. Even when surgery relieved pain and improved self-reported function, objectively measured activity often remained below the levels of healthy control populations at 6–12 months [75]. This mismatch helped motivate the shift toward richer digital phenotyping, including spatiotemporal gait parameters, asymmetry measures, cadence structure, and movement-quality metrics [1,5,27].

Subsequent reviews documented an increasingly broad set of wearable-derived outcomes, including stride length, speed, cadence, stance time, gait variability, range of motion, acceleration profiles, and symmetry measures across controlled testing and free-living settings [5,27]. Cadence-based measures such as peak six-minute consecutive cadence and peak one-minute cadence have been proposed as more stable and more discriminative indicators of recovery than total daily steps, with lower inter-day variability and earlier separation between hip and knee recovery profiles [32,73].

Large prospective cohorts using passive wearable data demonstrate that gait-quality recovery lags behind PROM improvement. In TKA, walking speed, step length, timing asymmetry, and double-limb support recover over roughly 13–24 weeks [12]. In THA, the same metrics generally recover earlier, within about 7–13 weeks [11]. Likewise, the largest Apple Watch cohorts show that steps and PROMs improve more rapidly than asymmetry, gait speed, and stair-related performance [9,10]. Together, these findings show that activity volume and movement quality should not be treated as interchangeable endpoints [9,10,11,12,13].

### 5.2. Advanced Gait Signal Processing

A smaller but increasingly important segment of the literature extracts higher-order features from raw accelerometer and gyroscope signals. Frequency-domain gait acceleration features derived from continuous thigh-mounted monitoring discriminated EQ-5D-3L responders from non-responders with AUC values of 0.80–0.81 after TKA [39]. Sample entropy analyses of trunk sway demonstrated systematic changes during recovery and correlated with conventional walking tests, suggesting that nonlinear signal complexity may function as a marker of movement normalization [40,45].

Single-sensor movement-quality frameworks have also been developed to characterize symmetry, smoothness, dynamic stability, and complexity across recovery. In TKA cohorts, these features deteriorated early after surgery, improved by 6 months, and correlated with patient-reported function [41,54]. Other studies used neural networks or support vector machines to predict spatiotemporal gait variables or classify impaired versus non-impaired gait, demonstrating that wearable data can support more refined functional phenotyping than step counts alone [51,52].

### 5.3. Population-Level Recovery Curves and Predictive Models

One of the strongest contributions of the wearable literature is the generation of empirical recovery curves. The largest multicenter cohorts provide benchmarks for typical recovery after THA and TKA across PROMs, step counts, gait asymmetry, gait speed, stair use, and steadiness [9,10,11,12,13]. These datasets show that recovery is multidomain and non-synchronous: in TKA, clinically important improvements in KOOS-JR, EQ-5D, and daily steps are concentrated around 3 months, whereas gait asymmetry improves later and stair-related function may continue to recover out to 12 months [9]. In THA, PROMs improve earlier, often within the first month, while objective mobility outcomes take longer to normalize or exceed baseline [10].

Predictive analytics is emerging from these longitudinal datasets. Machine-learning models using passive Fitbit data predicted subjective long-term recovery within 1 month of surgery, with performance that improved when longer preoperative baselines were available [72]. Logistic regression models based on IMU gait features tracked the probability that a patient still exhibited a TKA-like movement pattern over time [41]. At the same time, implant- and app-derived step counts show only moderate agreement in absolute values, even when they reflect similar broad recovery trajectories, underscoring that predictive models remain device- and metric-dependent [21].

## 6. From Monitoring to Personalization

### 6.1. Individual Baselines and Relative Recovery

A central idea in data-driven recovery is that postoperative progress should be interpreted relative to each patient’s own baseline rather than against a single universal threshold. Pilot studies collecting preoperative wearable data showed how quickly patients deviated from and then approached baseline values for step count, knee motion, and pain after TKA [47]. Similar work using passive consumer wearable data demonstrated that age, presurgical behavior, and surgery type influence recovery trajectories, and that models grounded in an individual’s presurgical baseline outperform approaches that rely only on postoperative data [34,72].

This baseline-centered approach is conceptually important because absolute step counts can conflate premorbid function with postoperative recovery. Longitudinal change more directly captures surgical impact, functional restoration, and delayed recovery. In smaller cohorts, changes in daily step count and minutes active were meaningfully associated with changes in physical health and HOOS/KOOS outcomes, further supporting relative rather than absolute interpretation [35].

### 6.2. Feedback, Behavior Change, and Remote Monitoring

Wearables can also function as behavioral tools rather than passive sensors. In a randomized trial, patients who received feedback from a Garmin Vivofit 2 and daily step goals achieved consistently higher postoperative step counts than those who wore the device without feedback [33]. Meta-analytic evidence supports this finding, showing that physical activity interventions incorporating wearable trackers are most effective when combined with behavior-change techniques and initiated within 2 weeks of surgery [14].

However, behavioral activation and clinical recovery are not equivalent. Although feedback interventions increase walking volume, they do not consistently improve PROMs over the same follow-up window [14,33]. Remote monitoring programs may nevertheless confer other advantages. In one pragmatic trial, a wearable-supported monitoring strategy did not improve activity levels or discharge destination, but it was associated with lower rehospitalization rates, implying that connectivity and surveillance may influence recovery through pathways other than step-count augmentation alone [36].

### 6.3. Structured Digital Rehabilitation Platforms

The most direct attempts at personalized rehabilitation are found in structured digital platforms that combine sensing, exercise prescription, progress feedback, and remote oversight. Studies by Correia et al. showed superior short- and medium-term outcomes for digital biofeedback systems after TKA and THA, including better Timed Up and Go performance and better KOOS subscale scores, although these studies were small, single-center, and quasi-randomized [59,60,61]. Subsequent work reported better early range of motion, lower manipulation-under-anesthesia rates, improved stiffness or pain subscales, and encouraging compliance in selected digital rehabilitation programs [23,46,55,58].

At the pooled level, smart device-assisted telerehabilitation is generally non-inferior to conventional rehabilitation with respect to pain and functional outcomes [15,30]. Yet subgroup analyses suggest that some modalities, particularly augmented reality, virtual reality, or motion-tracker-supported rehabilitation, may improve pain, knee extension, or selected functional endpoints [15,30]. The literature therefore supports digital rehabilitation as a credible alternative to standard care, but only selectively supports superiority [15,16,30,55,58,59,60,61]. The next step beyond describing recovery trajectories is to determine whether these data can be used to adapt rehabilitation to the individual patient.

## 7. Comparative Effectiveness and Clinical Integration

### 7.1. What Wearable-Supported Rehabilitation Improves

Across randomized trials and meta-analyses, the most consistent benefit of wearable-supported rehabilitation is improved step count [14,29,33,37,38]. Improvements in pain, function, and quality of life are more heterogeneous. Large syntheses conclude that digital rehabilitation usually achieves outcomes comparable to conventional physiotherapy across major clinical endpoints, with stronger signals of benefit for early range of motion, selected pain outcomes, and complication reduction [15,16,28,30,31,58,59,60]. Acute-care applications may also be relevant: smartphone-supported mobilization on postoperative day 1 increased standing and walking time and improved the odds of early functional recovery [22].

The implication is that wearable technologies are already clinically useful, but not because they have proven universal superiority over conventional rehabilitation. Their current value lies in extending observation beyond the clinic, improving adherence to walking goals, and identifying biomechanical features that conventional questionnaires do not capture [1,15,16]. Whether such monitoring improves care depends not only on technical accuracy, but also on whether wearable-supported pathways change outcomes, reduce complications, or integrate realistically into clinical workflows.

### 7.2. Accuracy, Adherence, and Workflow Barriers

Technical validity remains a major barrier to implementation. Consumer wrist-worn devices are unreliable when gait aids are used early after TKA [64]. Compliance estimates also change substantially depending on how a valid wear day is defined, which complicates comparison across studies and may exclude the very patients whose low mobility is most clinically important [65]. Different systems also generate systematically different absolute step counts, meaning that cross-device equivalence cannot be assumed [21].

Adherence and digital literacy add another layer of complexity. Dropout rates of 15–30% have been reported in digital intervention arms, and pairing issues, discomfort, and inconsistent wear contribute to missing data [26,59,60,61]. Nonetheless, several contemporary studies report high compliance and good patient satisfaction when digital pathways are well supported and feedback is intuitive [23,38,42,61].

System-level barriers are equally important. Reviews highlight concerns about data overload, clinician training, interoperability with electronic records, reimbursement, privacy, and inequitable access to proprietary device ecosystems [2,4,8,17,66,67,68,69,70,71]. Cost-effectiveness modeling has been favorable in at least one platform-specific analysis [70], but the evidence base remains concentrated in high-income settings, limiting immediate generalizability [66].

## 8. Discussion

### 8.1. Why Step Counts and PROMs Diverge

One of the most consistent findings in the literature is the dissociation between improved step counts and unchanged PROMs [14,33]. Several interpretations are plausible. PROMs may plateau before gait mechanics and activity quality have fully recovered [9,10]. The absolute increase in walking volume may remain below the threshold required for measurable symptom gains [33]. PROMs may also be limited by ceiling effects and subjective reporting bias, which make them less sensitive to objective improvements in capacity [1]. Finally, very early activity restraint may sometimes reflect an adaptive pain-management strategy rather than treatment failure [76].

### 8.2. Why THA and TKA Should Not Be Monitored Identically

The evidence strongly suggests that THA and TKA follow different recovery trajectories. THA patients recover gait-quality metrics earlier, and they display better walking steadiness than TKA patients even when step counts are similar [11,12,13]. Cadence-derived and gait-quality measures also separate hip and knee recovery profiles better than total daily steps [32]. These observations argue against generic monitoring pathways across procedures and support the development of surgery-specific recovery benchmarks and escalation thresholds [1,13].

### 8.3. Matching the Device to the Clinical Purpose

The literature does not support a simple consumer-versus-clinical device hierarchy. Instead, it supports contextual appropriateness. Consumer devices are well suited to long-term activity tracking, patient engagement, and large-scale recovery surveillance. Clinical-grade IMUs and rehabilitation-specific sensors are better suited to motion analysis, individual gait assessment, and decision support during the early postoperative period [1,27,64]. The future of personalized arthroplasty rehabilitation will likely depend on integrating these layers rather than selecting a single universal device class.

### 8.4. Evidence Quality and the Direction of the Field

Although the evidence base is now broad, it still includes many small observational and feasibility studies, especially in the gait-biomechanics literature [49,67]. The strongest data come from meta-analyses and large longitudinal cohorts, but the latter often lack randomized controls, and several platform-specific studies are closely tied to industry ecosystems [1,9,10,11,12,14,15,29]. Even so, the field has clearly evolved from simple pedometer-style activity monitoring to multidimensional phenotyping, prediction modeling, and digitally enabled rehabilitation [8].

The key unresolved question is whether wearable signals are being used to modify treatment in a prespecified, reproducible, and patient-specific way. Current evidence shows excellent progress in monitoring and characterization, and credible evidence for non-inferior telerehabilitation, but limited direct evidence that continuous wearable data are systematically translated into real-time individualized treatment decisions [1,2,14,15,16,17]. That distinction marks the boundary between remote observation and true data-driven personalization.

### 8.5. Limitations of This Review

This structured narrative review has several methodological limitations. It did not include formal risk-of-bias assessment, quality-based exclusion, quantitative pooling, or evidence grading. Device type, sensor placement, outcome definitions, wear-time thresholds, and rehabilitation models were heterogeneous. Primary studies were synthesized alongside reviews, validation work, and selected implementation papers, which supports a broad clinical account but restricts causal and comparative claims. In addition, 160 full-text records could not be accessed; excluding so many potentially eligible reports may have introduced selection bias.

## 9. Conclusions

Commercial wearables now provide real-world measures of recovery after THA and TKA that complement PROMs. The literature already supports step-count and adherence monitoring, procedure-specific recovery trajectories, and targeted assessment of gait quality or range of motion. Digital rehabilitation generally achieves outcomes comparable to conventional rehabilitation, with possible advantages for selected early outcomes.

Current evidence is stronger for measurement and remote follow-up than for automated personalization. Consumer devices are suited to scalable activity monitoring, whereas clinical IMUs are better for detailed biomechanical assessment. The next clinical step is to test alert systems that compare an individual trajectory with procedure-specific thresholds and trigger a predefined response. Until such pathways are prospectively validated, wearable data should inform, rather than independently determine, rehabilitation decisions.

## Figures and Tables

**Table 1:** Thematic overview of the included evidence base.

Evidence Domain	Representative Citations	Main Contribution	Implication for Personalized Recovery
Broad evidence syntheses	[1,2,3,4,5,6,7,14,15,16,17,24,25,26,27,28,29,30,31]	Reviews summarize device types, rehabilitation models, and outcome trends while highlighting methodological heterogeneity.	They define the current landscape and clarify where evidence is strong, weak, or inconsistent.
Large recovery-curve cohorts	[9,10,11,12,13,21,32]	Prospective datasets map recovery across PROMs, steps, gait quality, stair use, and steadiness over weeks to months.	They provide benchmarks for surgery-specific recovery trajectories.
Consumer tracking and behavior-change studies	[22,33,34,35,36,37,38]	Commercial trackers and apps can increase walking activity and support remote follow-up.	These approaches are scalable and useful for goal setting, but activity gains do not always translate into symptom improvement.
Clinical-grade gait and IMU studies	[18,19,20,23,39,40,41,42,43,44,45,46,47,48,49,50,51,52,53,54,55,56,57]	Higher-fidelity sensors quantify symmetry, speed, range of motion, variability, and movement quality.	These systems are most relevant for detailed biomechanical phenotyping and targeted monitoring.
Digital rehabilitation platforms	[23,46,55,58,59,60,61,62,63]	Sensor-assisted systems combine monitoring with exercise prescription, feedback, and remote oversight.	They represent the most direct route from measurement toward actionable rehabilitation decisions.
Validation and implementation studies	[2,4,64,65,66,67,68,69,70,71]	These studies address device accuracy, wear-time compliance, adoption barriers, workflow integration, and cost-effectiveness.	They determine whether wearable signals are sufficiently valid and feasible for routine care.

## Data Availability

No new data were created or analyzed in this study. Data sharing is not applicable to this article.
